# From EM to AI: Multiscale imaging of the secretory pathway with insights from plant cells

**DOI:** 10.1111/jmi.70115

**Published:** 2026-05-13

**Authors:** Charlotte Pain, Emily Farley

**Affiliations:** ^1^ Endomembrane Structure and Function Research Group, School of Biological and Medical Sciences Oxford Brookes University Headington Oxford UK; ^2^ Oxford Brookes Centre for Bioimaging Oxford Brookes University Headington Oxford UK

**Keywords:** artificial intelligence, endoplasmic reticulum, Golgi bodies, microscopy

## Abstract

The secretory pathway is a central and evolutionarily conserved feature of eukaryotic cells, responsible for protein and lipid trafficking, membrane biogenesis, signalling, and cellular homeostasis. Its complexity, dynamic behaviour, and nanoscale organisation have made it a longstanding target of microscopy‐driven investigation. In this review, we trace the parallel evolution of our understanding of the secretory pathway and imaging technologies, with a particular emphasis on plant cells, where unique architectural and functional features have challenged and enriched mechanistic models. We highlight the foundational role of electron microscopy (EM) in defining the ultrastructural organisation of secretory organelles and establishing directional models of intracellular transport, followed by the fluorescence microscopy revolution that enabled direct visualisation of cargo flux and organelle dynamics in living cells. The advent of super‐resolution fluorescence techniques bridged the long‐standing resolution gap between light microscopy and EM, revealing nanoscale compartmentalisation, membrane contact sites, and trafficking intermediates previously inaccessible in living cells. More recently, the integration of functional assays, optogenetics, and artificial intelligence‐driven segmentation, denoising, and adaptive imaging now enables quantitative and high‐throughput analysis of secretory architecture. Together, these advances have transformed the secretory pathway from a static morphological concept into a dynamic, increasingly mechanistically defined system. We conclude by discussing emerging integrative strategies, particularly correlative and AI‐enhanced approaches that promise to unify ultrastructural precision with molecular specificity and temporal resolution in future studies of endomembrane organisation.

## LAY DESCRIPTION

Cells rely on an internal delivery network called the secretory pathway to move proteins and fats to the correct locations, build membranes, send signals, and, in plants, construct the cell wall. Because many parts of this system are extremely small and constantly moving, scientists have depended on powerful microscopes to understand how it works. In this review, we describe how advances in microscopy have shaped our knowledge of the secretory pathway, particularly in plant cells. Early electron microscopes provided the first detailed images of structures such as the endoplasmic reticulum and the Golgi apparatus, key landmarks in the route taken by newly made proteins inside cells. However, these images were static snapshots. Fluorescence microscopy later allowed researchers to label specific proteins with glowing tags and watch them move inside living cells. This revealed that the secretory pathway is highly dynamic, with organelles and cargo in continuous motion. More recent super‐resolution techniques have uncovered tiny subregions within organelles and short‐lived transport carriers that were previously invisible. Today, artificial intelligence‐based image analysis can automatically identify organelles, measure their behaviour, and analyse large datasets. Together, these approaches provide a clearer, multi‐scale picture of how cells organise and regulate the movement of essential materials.

## 1 INTRODUCTION

**FIGURE 1:**
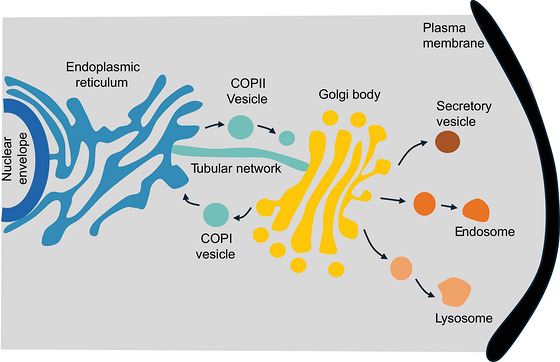
Summary of the eukaryotic secretory system. A brief overview of the organelles making up the secretory system. Newly synthesised proteins undergo folding, modification and quality control in the ER, and are transported via the Golgi body to their destinations. This can include the plasma membrane, endosomes, or lysosomes. Transport between the ER and Golgi has been hypothesised to occur via either vesicles or tubules.

The secretory pathway comprises a conserved network of endomembrane structures, including the endoplasmic reticulum (ER), the Golgi apparatus, transport and secretory vesicles, and the plasma membrane. Newly synthesised proteins enter the secretory pathway at the ER, where they are folded and packaged into transport vesicles for delivery to the Golgi apparatus. Following processing and sorting in the Golgi, cargo is trafficked via vesicles to its final destinations, including the plasma membrane, vacuole/lysosome, or extracellular space (Figure 1). These structures are thought to have evolved in the last eukaryotic common ancestor (LECA),^1^ underscoring their fundamental importance to cellular function. In mammalian and yeast systems, core functions of the secretory pathway have been described as protein folding, quality control, and post‐translational modification,^2^ lipid synthesis,^3^ and metabolite storage.^4^, ^5^ However, this overview represents only a fraction of the pathway's full functional repertoire (Table 1), with more than 30% of mammalian proteins passing through the secretory system.^6^ Despite its evolutionary conservation and central role in cellular physiology, notable differences exist between mammalian and plant secretory systems.

**TABLE 1:** **Key functions of the secretory pathway**. Commonly discussed functions of the components of the secretory pathway or the secretory pathway as a whole, alongside key references.

Secretory pathway function	Description	Key/example references
Protein folding and quality control	Ensures newly synthesised proteins fold correctly and are checked before proceeding through the secretory pathway (ER quality control).	^2^, ^7^
Post‐translational modification	Modifications like glycosylation and processing in the ER and Golgi influence protein stability and function.	^8^, ^9^
Lipid synthesis and membrane assembly	Secretory organelles, especially ER and Golgi, contribute to lipid biosynthesis and membrane formation.	^3^, ^10^
Hormone and growth factor secretion	Storage and regulated release of peptide hormones and growth factors via secretory granules.	^11^, ^12^
Cell polarisation	Spatially control the deposition/localisation of key proteins to set up gradients across the cell.	^13^, ^14^
Calcium regulation	Calcium stored/transported in secretory organelles influences vesicle fusion and signalling.	^15^, ^16^
Cellular homeostasis	Balanced protein/lipid transport maintains organelle and cellular equilibrium.	^17^
Cell‐cell communication	Delivers signalling molecules to the membrane or extracellular space to communicate between cells.	^18^, ^19^
Lysosome/vacuole targeting and degradation	Routes specific enzymes to lysosomes/vacuoles for digestion and turnover.	^20^
Plant defence and immune responses	The pathway actively traffics defence proteins, receptors, and wall components to counter pathogens.	^21^, ^22^
Plant cell wall component synthesis	Supplies enzymes and carriers for plant cell wall biosynthesis and deposition.	^23^, ^24^

In plants, the presence of a large vacuole,^25^ highly dynamic organelle behaviour,^26^, ^27^ and alternative endosomal organisation confer unique functional characteristics.^28^, ^29^, ^30^ Divergent functions are also observed in fungi, where the secretory pathway plays essential roles in polarised cell growth and hyphal elongation, with secretory vesicles accumulating at the Spitzenkörper, a vesicle supply centre at the hyphal tip, to direct localised exocytosis.^31^ Additionally, fungal cell wall biosynthesis is dependent on the delivery of cell synthases to the plasma membrane, which is undertaken by the secretory pathway.^32^ Despite its evolutionary conservation and central role in cellular physiology, notable differences exist between mammalian, plant, and fungal secretory systems, reflecting their distinct cellular architectures and functional demands. In plants, the presence of a large vacuole,^25^ highly dynamic organelle behaviour,^26^, ^27^ and notably the non‐perinuclear distribution of endomembrane organelles distinguish their secretory organisation from mammalian systems. This spatial separation results in discrete, motile Golgi stacks distributed throughout the cytoplasm, making plant cells particularly amenable to microscopy‐based analysis of secretory dynamics.

**FIGURE 2:**
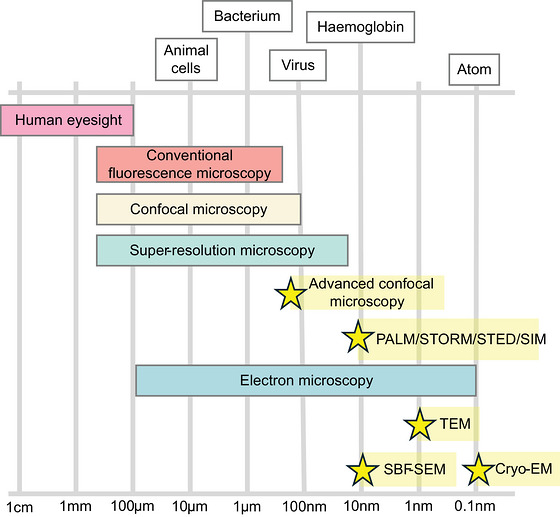
The approximate size of different cellular structures alongside the resolution of common microscopy techniques. Comparison of the available resolution of numerous microscopy techniques, such as confocal microscopy and electron microscopy, alongside commonly observed biological structures, such as bacteria and viruses. Also pinpointed with yellow stars are the best‐reported X/Y resolutions of specific techniques, including PALM/STORM and Cryo‐EM.

The secretory pathway presents an ideal system for microscopy‐based studies, particularly as many endomembrane components exhibit overlapping content and structural component distributions.^33^ For instance, the outer nuclear envelope is fully contiguous with the ER, making separation of the two distinct structures particularly challenging without the use of imaging techniques. This problem can be compounded by proteins that have overlapping spatial distributions, for example, in mammalian cells, Peptidylglycine α‐amidating monooxygenase (PAM), which can localise to both the plasma membrane and the trans‐Golgi network.^34^ A spatial overlap of biochemical identity is particularly pronounced within Golgi compartments in mammalian and plant cells, where glycosylation enzymes frequently display at least partially overlapping distributions.^35^, ^36^, ^37^ The study of the secretory pathway also favours a multimodal imaging approach, particularly as many components of the secretory pathway are at or near the diffraction limit of light, approximately 200–250 nm laterally. For example, COPII vesicle size has been reported at approximately 60–80 nm in diameter in yeast and mammalian cells^38^, ^39^ while the width of ER tubules has been reported at as low as 40 nm in plant cells.^40^ This places the size of key secretory pathway organelles at the limits of conventionally used optical microscopy, but well within the limits of electron microscopy (Figure 2). Though it would therefore seem that electron microscopy (EM) would be the ideal imaging modality for the study of the secretory pathway, the components of the pathway remodel rapidly as part of their function.^27^, ^41^, ^42^ This rapid remodelling cannot be observed using EM techniques due to the need for sample fixation and staining with heavy metals, making the secretory pathway a particularly suitable target for multimodal imaging approaches.

## 2 INITIAL IDENTIFICATION OF SUBCELLULAR COMPONENTS USING LIGHT MICROSCOPY

The components of the secretory pathway were first identified by optical microscopy, with ‘cells’ first described by Hooke in 1655^43^ and the first subcellular structures described on the turn of the 17th century, primarily in mammalian cells.^44^ The first conventional descriptions and naming conventions of cellular structures were made in the 1830s. This work began with the identification of the nucleus, followed by mitochondria, and then by the ER (initially described as ergastoplasm).^45^, ^46^, ^47^, ^48^ These organelles were often first observed without stains, with researchers typically relying on changes to cellular content density which occurred due to the presence of different subcellular structures. Confirmation of their identities was typically based on more recently developed microscopy stains. Light microscopy is necessarily limited in its maximum resolution by the diffraction limit of light; modern diffraction‐limited confocal systems approach approximately 200 nm lateral resolution,^49^ some super‐resolution techniques reach significantly lower reported resolutions. For example, SIM (structured illumination microscopy) has been reported as reaching resolutions of 100–150 nm,^50^, ^51^ STED (stimulated emission depletion) can reach 40–80 nm^52^, ^53^ and single‐molecule imaging techniques such as STORM (stochastically optical reconstruction microscopy) and PALM (photoactivated localisation microscopy), can reach 20–40 nm (Figure 2).^54^, ^55^, ^56^, ^57^ However, reaching these lower resolutions comes with significant experimental drawbacks, including the need for high laser power requirements of STED,^58^ which can cause phototoxicity in many samples. Meanwhile, PALM/STORM is a speed‐limited technique with strict limits on the appropriate fluorophore selections.^58^ These vast improvements in the resolution of optical microscopy techniques have come very recently, between the initial identification of secretory pathway organelles by optical microscopy and the recent advances of super‐resolution optical microscopy, came the golden age of electron microscopy.

## 3 THE GOLDEN AGE OF ELECTRON MICROSCOPY: BUILDING A LASTING FOUNDATION

The development of the modern electron microscope began with the invention of the cathode ray tube, which Ernst Ruska, Bodo von Borries and Max Knoll used to create the first two‐stage transmission electron microscope (TEM) with a magnetic lens of magnification 13× in 1931.^59^, ^60^ After years of development, Ernest Ruska improved their original TEM to include three magnetic lenses, including one acting as the condenser and the other two as the objective and projection lenses. This microscope allowed for 12,000×, and a camera located outside the vacuum could photograph the magnified area. This would eventually be sold as the first commercial TEM, manufactured in the UK.^61^ Following the invention of TEM, Manfred von Ardenne developed the Scanning electron microscope (SEM) in 1938, allowing images of sample surfaces to be obtained at a high magnification and resolution.^62^ These two techniques are still widely used in biological research today.

The advent of electron microscopy marked a significant milestone in both the discovery of the secretory pathway and in the life sciences. However, early implementations of TEM were limited by the lack of reliable ultrathin sectioning techniques, restricting observations to whole mounts or relatively thick specimens with poor intracellular resolution. The subsequent development of ultramicrotomy, enabling the preparation of sections typically <100 nm in thickness, was therefore critical for resolving internal membrane‐bound organelles such as the endoplasmic reticulum and Golgi apparatus.^63^ Despite this advance, the requirement for chemical fixation, dehydration, embedding, and sectioning introduces potential artefacts, including distortions of membrane morphology and loss of dynamic context.^64^ In particular, the two‐dimensional nature of ultrathin sections can complicate structural interpretation, as tubular membranes may appear as discrete vesicular profiles depending on the plane of section. In contrast, scanning electron microscopy requires different sample preparation strategies, typically involving surface fixation, dehydration, and conductive coating, and provides detailed three‐dimensional information on cell surfaces rather than internal ultrastructure.^65^ While SEM has proven valuable for examining cell morphology and surface‐associated secretory events, it is inherently less suited to resolving the internal organisation of the secretory pathway, for which TEM remains the principal approach despite its limitations.

One of the most well‐regarded early studies using EM was the publication of an intact cell taken by Albert Claude, Keith Porter and Ernest Fullam in 1945.^66^ The image magnified a mammalian fibroblast to 1600 times its original size and revealed the structure of the mitochondria, the Golgi body and a structure that was later renamed by Porter as the ‘endoplasmic reticulum’.^66^ Notably, the Golgi apparatus was first described by Camillo Golgi in 1898 using a silver staining method,^67^ which revealed what he termed the ‘internal reticular apparatus’ in neurons. This observation, made using light microscopy, was initially met with considerable scepticism, with many researchers proposing that the structure represented a staining artefact rather than a genuine cellular component.^68^ It was not until the advent of electron microscopy in the 1950s, with the publication of over 70 further articles studying its physiology and localisation across plant, mammalian and yeast cells,^69^ that the existence and ultrastructural organisation of the Golgi apparatus were definitively confirmed. It is now known to be composed of multiple, stacked, self‐enclosed membranes surrounded by several smaller circular vesicles.

Early images of the Golgi body were captured using buffered osmium tetroxide as a cellular stain.^70^ This was a key technological development, as in order for electron microscopy to be broadly adopted by the life sciences, parallel developments of electron‐dense stains were required to improve the visualisation of biological samples. To capture images by electron microscopy, targeted electrons must interact with the given sample. As biological samples are typically carbon‐based, they only weakly interact with electrons due to their sparse nuclei, resulting in poorly contrasted images. To combat this issue, heavy metal salts such as lead, osmium and uranium were used as stains.^71^ Palade's introduction of buffered osmium tetroxide markedly improved the preservation of membranes and paved the way for modern EM fixation.^72^ In the 1950s, aldehyde fixatives, for example, formaldehyde and glutaraldehyde, were developed for EM and provided significantly better ultrastructure preservation. Similarly, embedding techniques were developed with the use of epoxy resins, which were much more stable than earlier plastic resins, allowing for ultrathin sectioning with ultramicrotomes.^73^ Eventually, during the 1960s–1970s, a sample protocol using uranyl acetate and lead citrate had been developed, following a pattern of first fixation by an aldehyde, followed by osmium, uranyl and lead, which is still used widely to image cells and tissues.^74^

The concept of the secretory pathway, and the order of organelles through which secreted proteins must pass, was first conceived of by integrating EM with improved cell fractionation and autoradiography. Early experiments were able to trace secretory proteins within the mammalian exocrine pancreas from their site of synthesis on the rough ER, through distinct Golgi elements, to structures they termed ‘condensing vacuoles’, and ultimately into zymogen granules before secretion.^75^ Electron microscopy was essential to this work, as it revealed the spatial continuity and compartmental communication of organelles underlying secretion. This landmark study not only established the secretory pathway as a core framework in cell biology but also demonstrated the power of EM to link cellular ultrastructure with dynamic biological processes. Importantly, these pulse‐chase autoradiography studies did more than map a trafficking route: they established a directional, compartmentalised model of secretion in which cargo moved sequentially from the ER to *cis‐*, medial‐, and *trans*‐Golgi cisternae before packaging into secretory granules.^75^ However, this interpretation also reinforced a vesicle‐centric view of transport that dominated the field for decades. Subsequent work questioned whether Golgi cisternae themselves mature and progress forward, rather than remaining static while cargo is shuttled between them.^76^, ^77^ Thus, even at its inception, the EM‐defined secretory pathway contained the seeds of an enduring mechanistic debate: vesicular transport versus cisternal maturation. This debate was, in part, shaped by the intrinsic limitations of electron microscopy, which provides high‐resolution but static snapshots of fixed material; such images can lead to misinterpretation of morphology, for example, tubular profiles may appear vesicular in thin sections, and do not convey the directionality of membrane flux, obscuring whether observed structures represent budding or fusion events. Microscopy did not merely reveal structure but shaped the conceptual models that structured interpretation for generations. Subsequent studies built upon this EM‐defined foundation to dissect the molecular mechanisms of secretion, including in vitro reconstruction approaches by Günter Blobel and later genetic and biochemical strategies developed by James Rothman and Randy Schekman in mammalian cells.^78^, ^79^

As the resolution of the microscopy techniques used to study the secretory pathway improved, there developed a greater understanding of the diversity of the secretory pathway across species. One of the most referenced schisms is the differences between mammalian and plant cells. For example, plant cells typically contain numerous distinct Golgi bodies, as opposed to a singular, larger Golgi body observed in mammalian cells.^80^ Due to the unexpected differences between plant and mammalian Golgi bodies, plant Golgi bodies were even given a specific name ‐ ‘dictyosomes’ (originally used to describe fragments created when the Golgi undergoes mitosis^81^). In 1964, Morré and Mollenhauer provided the first isolation of intact plant dictyosomes or Golgi, which enabled ultrastructural studies and biochemical characterisation.^82^ Morré and Mollenhauer developed biochemical and ultrastructural approaches that established the plant Golgi apparatus as a tractable system for functional analysis beyond descriptive morphology. They introduced protocols for isolating Golgi membranes and stabilising them with glutaraldehyde, enabling examination of cisternal architecture and associated vesicles by negative staining and electron microscopy, and facilitating subsequent biochemical assays of enzyme activities and lipid composition in purified fractions.^83^ These methodological advances provided important structural context for understanding functional specialisation within dictyosome cisternae and informed early models of cargo processing and vesicle formation in plant secretion.^83^

Concurrently, autoradiographic tracing experiments in root‐cap cells demonstrated that newly synthesised polysaccharides labelled with radiolabelled glucose move from Golgi stacks into associated vesicles and across the plasma membrane into the cell wall.^84^, ^85^, ^86^ Collectively, these combined ultrastructural and biochemical observations supported the concept that the Golgi apparatus in plant cells is centrally involved in the synthesis, modification, and export of polysaccharide components of the cell wall, directly linking organelle organisation with secretory function.

## 4 THE RISE OF FUNCTIONAL EM

The integration of biochemical assays with electron microscopy established what is now referred to as functional electron microscopy, enabling direct linkage between ultrastructural organisation and biosynthetic activity within the secretory pathway. In plant cells, this approach proved particularly powerful for dissecting the synthesis and export of cell wall components. By combining subcellular fractionation with EM‐based validation of organelle purity, functional EM enabled the assignment of specific biosynthetic activities to distinct Golgi membranes and trafficking intermediates. These studies demonstrated that polysaccharides synthesised in the Golgi are packaged into vesicles and exported to the plasma membrane for cell wall deposition, providing a structural and biochemical framework for secretory flux through the Golgi apparatus.^85^, ^86^

Extending these observations, enzymatic assays and ultrastructural analyses revealed that polysaccharide and glycoprotein biosynthesis are spatially organised within the Golgi stack. Distinct glycotransferase activities were mapped to different Golgi regions, supporting a model in which cargo undergoes sequential modification as it progresses through cisternal compartments.^87^, ^88^ This work provided early experimental evidence for functional compartmentalisation within the Golgi and laid the groundwork for later cisternal maturation models. Yet, despite increasing biochemical evidence for cisternal specialisation, electron microscopy alone could not resolve whether Golgi compartments were stable entities or dynamic structures undergoing continual renewal. Serial sectioning and enzyme localisation supported ordered organisation,^36^, ^87^ but static images could not distinguish between competing transport models. This limitation illustrates a broader principle: ultrastructural precision does not automatically confer mechanistic clarity. Only through subsequent integration with live‐cell fluorescence imaging and genetic perturbation did it become possible to test whether cisternae themselves progress, mature, or exchange components dynamically.^89^, ^90^ Thus, functional EM established structural compartmentalisation but left key kinetic questions unresolved. In parallel, functional EM approaches were applied to investigate Golgi membrane dynamics and protein transport mechanisms. Serial sectioning and enzyme‐labelled EM analyses demonstrated continuity between the ER, Golgi membranes, and the plasma membrane, supporting a unified membrane flow model of secretion. These plant cell studies established that Golgi cisternae are dynamic structures derived from ER membranes rather than static entities, highlighting the role of continuous membrane flux in sustaining secretory pathway function.^91^, ^92^

Confidence in the data produced by EM studies has increased with the development of rapid fixation techniques that are designed specifically to improve membrane preservation during fixation.^93^ Building on these advances, high‐pressure freezing (HPF) enabled the capture of near‐native cellular architecture, facilitating the visualisation of transient and highly dynamic membrane structures, overcoming the limitations of chemical fixation.^94^ Subsequent developments in three‐dimensional EM, particularly electron tomography, extended these capabilities by resolving organelle connectivity and membrane organisation in situ.^95^ Tomographic reconstructions in the 2000s captured ER exit sites, trans‐Golgi network organisation, and extensive cisternal tubulation, offering quantitative and spatial resolution unattainable in conventional 2D EM.^96^ Collectively, these methodological innovations established electron microscopy as a decisive force in defining the structural and biochemical organisation of the secretory pathway.

## 5 CRYO‐ELECTRON MICROSCOPY AND IN SITU STRUCTURAL BIOLOGY

While earlier advances in fixation and electron tomography substantially improved ultrastructural preservation, concerns regarding artefacts and membrane distortion remained.^97^ Cryo‐electron microscopy (cryo‐EM) and cryo‐electron tomography (cryo‐ET) addressed these limitations by enabling imaging of vitrified biological material in a near‐native, hydrated state, avoiding chemical fixation, dehydration, and staining. Cryo‐electron tomography further enabled three‐dimensional reconstruction of intact cellular regions at nanometre resolution, allowing visualisation of membrane contact sites, vesicle coats, and macromolecular assemblies directly in situ.^98^, ^99^ Within the secretory pathway, cryoET has resolved the architecture of COPII coats at ER exit sites and clarified coat assembly intermediates,^100^, ^101^ refining mechanistic models of vesicle formation and cargo packaging.

Notably, tomographic datasets frequently reveal tubular continuities and closely apposed membrane networks between ER and Golgi compartments^102^ challenging exclusively vesicle‐centric models of ER‐Golgi transport and supporting alternative frameworks such as cisternal maturation and tubular transport. Although cryo‐ET captures static snapshots, the preservation of transient intermediates provides critical insight into the structural states underlying dynamic trafficking.

Despite these advances, cryo‐EM approaches remain technically demanding, particularly in plant systems where cell walls necessitate high‐pressure freezing and cryo‐focused ion beam milling.^94^, ^103^ Throughput remains limited compared to fluorescence microscopy, and molecular identity is often inferred rather than directly visualised. Correlative light and electron microscopy (CLEM) has emerged as a powerful approach to bridge ultrastructural information with fluorescence‐based protein localisation, enabling molecular identity to be assigned to defined membrane compartments.^104^, ^105^, ^106^ These integrative strategies bridge structural and dynamic analyses of the secretory pathway, but the inability of cryo‐EM to capture real‐time dynamics continues to drive the development of fluorescence‐based approaches capable of resolving trafficking behaviour in living cells.

## 6 THE FLUORESCENCE REVOLUTION: VISUALISING THE SECRETORY PATHWAY IN LIVING CELLS

A critical step in the evolution of microscopy applied to the secretory pathway was the development of protein‐specific labelling strategies in fixed samples. Prior to the advent of genetically encoded reporters, the ability to localise individual proteins within the ultrastructural context of the cell relied heavily on immunostaining approaches using labelled primary or secondary antibodies. In light microscopy, immunofluorescence enabled the visualisation of secretory pathway components, such as ER‐ and Golgi‐resident proteins, with increasing specificity, thereby transforming what had been largely morphology‐driven interpretations into molecularly defined maps of intracellular organisation.^107^, ^108^ This approach was particularly powerful when combined with chemical fixation protocols that preserved subcellular architecture, allowing researchers to correlate protein localisation with organelle identity.

Despite their strengths, these antibody‐based labelling methods were inherently limited to fixed specimens, precluding direct observation of dynamic processes such as vesicle budding, trafficking, and fusion. The subsequent introduction of genetically encoded fluorescent proteins, most notably green fluorescent protein (GFP), marked a paradigm shift by enabling protein‐specific labelling in living cells.^109^, ^110^ However, the conceptual and methodological foundations established by immunostaining and immuno‐EM were essential for interpreting these dynamic imaging experiments. Early GFP‐based studies of the secretory pathway, for example, often relied on prior knowledge derived from fixed‐cell immunolabelling to validate localisation patterns and infer functional behaviour.

The transition from static to dynamic cell imaging of the secretory pathway began in earnest with the rise of confocal microscopy, enabled by technological developments in laser systems and computational image processing. This allowed for the real‐time observation of secretory structures and processes in living cells, which previously could only be inferred from fixed samples. However, live‐cell imaging is not without its own experimental constraints: the illumination required to excite fluorophores can induce photobleaching and phototoxic effects, generating reactive oxygen species and perturbing cellular physiology, particularly during prolonged imaging or at high laser intensities. As a result, a balance must be struck between spatial‐temporal resolution and the preservation of normal cellular function. Within these constraints, the coupling of fluorescent protein tags to components of the secretory pathway revolutionised real‐time tracking of organelles and cargo flows, enabling significantly improved mechanistic insights into secretory pathway dynamics.

**FIGURE 3:**
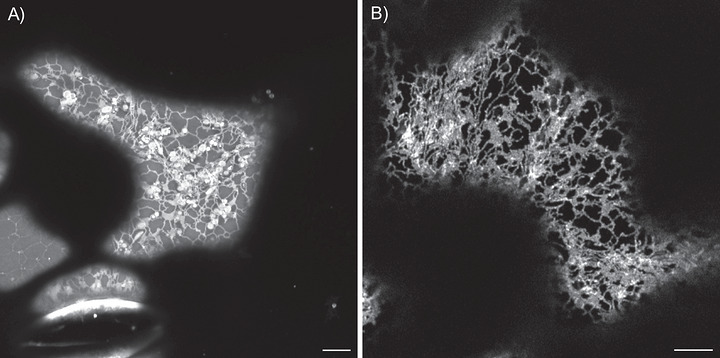
Comparison of the use of a lipid dye and a target GFP to observe the endoplasmic reticulum in plant cells. The endoplasmic reticulum (ER) of (a) 10‐day‐old Arabidopsis cotyledon cells stained with rhodamine B to show the ER and mitochondria, compared to (b) the ER in a tobacco leaf epidermal cell 3 days after undergoing agrobacterium‐mediated transformation with GFP‐HDEL. Images were collected on a Zeiss 880 LSM confocal with an Airyscan detector. Image was post‐processed for Airyscan. Scale bars 5 µm.

The development of GFP (green fluorescent protein) as a genetically encoded fluorescent protein that could be manipulated for biological studies was essential for real‐time visualisation of secretion within cells.^109^, ^110^ Although numerous fluorescent dyes, such as FM1‐43, rhodamine B (Figure 3) and BODIPY derivatives, have been successfully deployed to image the secretory pathway, they come with increased risks of altered membrane dynamics, perturbed cellular structures and cytotoxicity.^111^, ^112^, ^113^ This is partly because many fluorescent dyes are lipophilic and partition into lipid bilayers, where their localisation is governed by passive diffusion, membrane affinity and electrochemical gradients rather than strict molecular specificity. Consequently, these probes can redistribute between membrane compartments and diffuse laterally within bilayers, which may perturb membrane organisation and complicate interpretation of ‘specific’ organelle labelling.^114^ This can particularly be the case in studies involving cell types with poor permeability to dyes, for example, plant and fungal cells with thick cell walls, which can require the addition of detergents to aid dye penetration into in the cell.^115^, ^116^ GFP however, allows for in situ tagging of specific proteins within living cells, with minimal perturbations to cellular structures (as confirmed by EM‐based studies). GFP was first isolated from *Aequorea victoria* in 1962^117^ but truly emerged as a central tool in molecular biology through molecular cloning^109^ and subsequent structural engineering. The enhanced GFP (eGFP) variant exhibited improved brightness, photostability, and compatibility with standard laser lines.^118^ The expansion of the fluorescent protein palette, including red variants derived from *Discosoma*,^119^ enabled multicolour imaging of proteins and organelles. Adaptation of GFP for plant systems, including removal of a cryptic intron, further extended its applicability.^120^ Live‐cell imaging revealed the highly motile nature of the ER and Golgi apparatus. In plant cells, GFP‐HDEL, GFP retained in the ER by a minimal localisation signal, demonstrated ER continuity, cytoskeletal coupling, and the dynamic association of Golgi bodies with ER membranes.^42^ Subsequent studies established actomyosin‐dependent Golgi motility and introduced trafficking assays such as secGFP, enabling functional interrogation of ER‐Golgi transport.^121^, ^122^, ^123^

Fluorescence recovery after photobleaching (FRAP) demonstrated ATP‐dependent protein exchange between the ER and Golgi,^124^ while photoactivatable GFP revealed directed protein flow exceeding diffusion‐based expectations.^125^ Protein–protein interactions were resolved using Förster resonance energy transfer coupled with fluorescence lifetime imaging microscopy (FRET‐FLIM),^126^, ^127^ enabling nanoscale interaction mapping in living plant cells. This approach elucidated compartment‐specific glycosylation complexes and vesicle tethering mechanisms within the Golgi apparatus within plant cells.^128^, ^129^ Despite these advances, confocal microscopy remained limited by the diffraction barrier (∼200–250 nm), leaving many subcellular structures unresolved. Live‐cell fluorescence imaging fundamentally altered the interpretation of secretory pathway organisation by revealing that organelles once thought to be relatively static are in fact highly dynamic and continuously exchanging components. In plant cells, the discovery of motile Golgi stacks moving along actin filaments directly challenged mammalian‐centric architectural assumptions.^42^, ^121^ Moreover, kinetic measurements of cargo flux and protein turnover began to support cisternal maturation models in which resident enzymes recycle retrogradely while cisternae progress anterogradely.^130^ However, fluorescence microscopy introduced new interpretive challenges: overexpression of tagged proteins can perturb trafficking dynamics, and diffraction‐limited resolution complicates assignment of proteins to discrete subcompartments.^131^ Thus, while live imaging resolved temporal ambiguity inherent to EM, it introduced new sources of experimental uncertainty. This gap motivated the adoption of super‐resolution fluorescence techniques, including STED, SIM, STORM, and PALM.^56^, ^57^, ^132^

**FIGURE 4:**
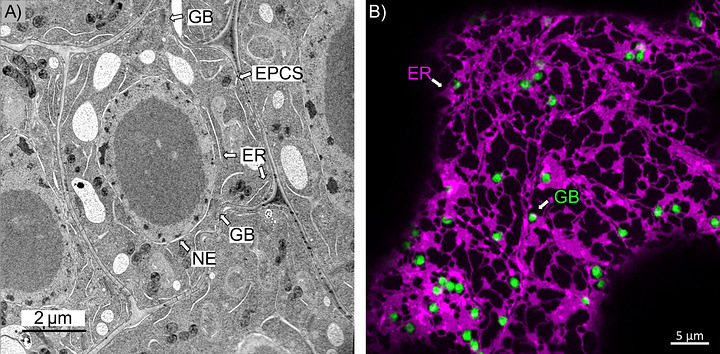
Comparison of cellular structures observable using EM and confocal microscopy. Representative images of (a) an Arabidopsis root cell and (b) a tobacco leaf epidermal cell. The Arabidopsis roots were fixed using HPF, followed by osmium tetroxide and uranyl acetate staining. The image was collected on a JEOL JEM‐1400Flash (JEOL) transmission electron microscope. The tobacco epidermal cell is a live‐cell transiently expressing the ER marker RFP‐HDEL (magenta) and the Golgi body marker ST‐GFP (green). Images were collected on a Zeiss 880 LSM confocal with an Airyscan detector. Image was post‐processed for Airyscan. Key organelles are marked on the images for clarity, including Golgi bodies (GB), the endoplasmic reticulum (ER), ER‐plasma membrane contact sites (EPCS), and the nuclear envelope (NE).

Super‐resolution imaging revealed previously understudied features of the secretory pathway, including Golgi cisternal organisation, ER‐plasma membrane contact sites, and vesicle nanodomains. Commercial innovations such as Airyscan further democratised sub‐diffraction imaging by enhancing confocal resolution through detector arrays.^133^ More recently, Super‐Resolution Confocal Live Imaging Microscopy (SCLIM) integrated spinning‐disk confocal optics with optimised detection to achieve high spatial and temporal resolution in living plant cells. SCLIM studies revealed functional zonation within the trans‐Golgi network and defined cargo‐specific secretory vesicle clusters in mammalian cells.^134^, ^135^, ^136^ Nanoscale imaging also revitalised debate surrounding ER‐Golgi transport mechanisms. Super‐resolution and STED imaging resolved thin ER tubules closely connected to Golgi stacks, suggesting the presence of transient membrane continuities alongside vesicular transport.^137^, ^138^ Integration of super‐resolution imaging with functional assays, including FRAP, FRET‐FLIM, optogenetics, and single‐particle tracking PALM, has further refined understanding of SNARE organisation, membrane nanodomains, and retrograde trafficking pathways.^139^, ^140^, ^141^ Super‐resolution imaging has not simply refined spatial precision; it has re‐opened mechanistic questions previously considered settled. The visualisation of nanoscale compartmentalisation within the Golgi and trans‐Golgi network has revealed subdomains that challenge simplistic cis‐medial‐trans compartment models.^142^, ^143^ Similarly, high‐resolution imaging of ER‐Golgi interfaces suggest a spectrum of transport modalities, including vesicles, tubular continuities, and membrane contact sites.^144^ Rather than resolving longstanding debates outright, super‐resolution microscopy has exposed additional layers of complexity, highlighting that improved resolution often reveals new heterogeneity rather than definitive answers. Despite nanoscale advances, fluorescence methods alone cannot fully resolve membrane ultrastructure. This limitation motivated correlative strategies that directly integrate molecular identity with electron‐dense architecture (Figure 4).

The integration of super‐resolution techniques with live‐cell imaging represents a powerful but technically challenging frontier in the study of the secretory pathway. Methods such as STED, SIM, and single‐molecule localisation approaches (e.g., PALM/STORM) offer nanoscale spatial resolution, but their application to living systems is constrained by trade‐offs between resolution,^145^ imaging speed, and phototoxicity. High illumination intensities and repeated acquisition cycles, particularly in STED and localisation microscopy, can exacerbate photobleaching and induce cellular stress, limiting observation times and potentially perturbing trafficking dynamics.^146^ Conversely, approaches such as SIM provide improved temporal resolution and reduced photodamage, but at the cost of more modest gains in spatial resolution. Additional challenges include the need for highly photostable fluorophores, increased data processing demands, and the difficulty of maintaining physiological expression levels of tagged proteins during high‐resolution imaging.^147^ As a result, while live‐cell super‐resolution imaging has begun to reveal nanoscale dynamics of secretory compartments, its application often requires careful optimisation and remains best suited to targeted questions where the balance between spatial precision and physiological relevance can be tightly controlled.

## 7 CORRELATIVE LIGHT AND ELECTRON MICROSCOPY (CLEM): BRIDGING FORM AND FUNCTION

Correlative light and electron microscopy (CLEM) has emerged as a powerful integrative imaging strategy that combines the molecular specificity of fluorescence imaging with the ultrastructural resolution of electron microscopy. This is achieved by, on advanced systems, using both light and electron microscopy on the same instrument. In addition to integrated platforms, CLEM can also be performed using separate light and electron microscopes by imaging the same sample sequentially, with correlation achieved through fiduciary markers, patterned substrates, or intrinsic cellular landmarks that enable precise alignment of fluorescence and electron micrographs. Fluorescent markers and proteins are used to pinpoint the specific localisation of proteins within a sample, possibly during dynamic processes, while the use of electron microscopy provides significantly greater ultrastructural resolution of the underlying cellular components. The different advantages of each imaging system can be clearly observed when comparing example EM and confocal images (Figure 2). EM microscopy shows the fine details of cells, such as the complex ribbon structure of the plant Golgi body, while using conventional confocal microscopy, individual Golgi bodies can be specifically identified by specifically targeted fluorophores; they appear more as flat discs than complex, multilayered organelles.

**FIGURE 5:**
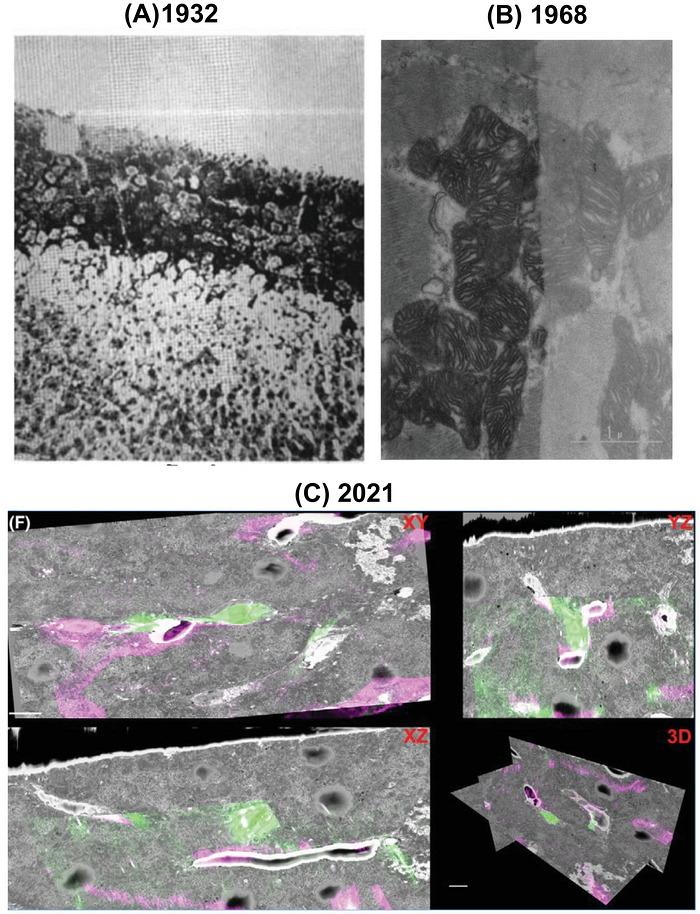
The evolution of microscopy capabilities. (a) Early images of guinea pig liver tissue stained with osmium tetroxide (magnification ×80) produced by Underhill.^148^ (b) Partially stained rat heart imaged using low voltage electron microscopy (osmium tetroxide, ×36,000, image collected and published by Hayden, 1968^149^ and (c) a modern CLEM image of a mouse liver with Kupffer cells in green and blood vessels in magenta. Scale bar 10 µm and image collected and published by Kremer et al.^150^

These capabilities are rooted in earlier fixation and staining approaches, where immunofluorescence and immuno‐electron microscopy first enabled proteins to be localised within defined cellular structures, albeit in static snapshots.^148^ The progression from these early antibody‐based methods to modern CLEM highlights the rapid evolution of microscopy, with successive advances converging to integrate molecular specificity, ultrastructural detail, and, increasingly, dynamic information within a single experimental framework (Figure 5).

In practice, the widespread use of CLEM is still limited due to several core challenges, including sample preservation, registration between imaging modalities and maintaining fluorescence throughout sample preparation.^104^, ^105^ However, despite these challenges, CLEM has been successfully applied to the exploration of the secretory pathway. Notably, CLEM has recently been used to characterise the formation of COPII vesicles in yeast and has provided insights into the structure and function of the ER‐Golgi interface.^106^, ^151^ Looking ahead, CLEM workflows will likely be strengthened by the continued development of fixation‐resistant fluorophores and improved image registration tools to align datasets rapidly across multiple modalities.

## 8 ARTIFICIAL INTELLIGENCE AND COMPUTATIONAL MICROSCOPY: QUANTITATIVE AND PREDICTIVE IMAGING OF THE SECRETORY PATHWAY

As imaging technologies have increased in resolution and speed, microscopy datasets have expanded in size and complexity, necessitating automated analytical approaches. Artificial intelligence (AI), particularly deep learning, has emerged as a transformative tool in biological image analysis.^152^, ^153^ Convolutional neural networks such as U‐Net^154^, ^155^ architectures enable robust segmentation of organelles in fluorescence and electron microscopy datasets across diverse systems including plant, yeast, and mammalian cells. In the context of the secretory pathway, AI‐assisted segmentation has facilitated quantitative analysis of ER network topology, Golgi stack morphology, and vesicle populations across large datasets, reducing observer bias and enabling statistically robust comparisons. Automated EM segmentation approaches^156^ have further enabled three‐dimensional reconstruction of organelle connectivity and membrane contact sites at scale. Beyond segmentation, deep learning‐based denoising and restoration algorithms improve signal‐to‐noise ratios while preserving structural detail, permitting reduced illumination intensities and minimising phototoxicity during live‐cell imaging.^157^ Computational super‐resolution methods extract sub‐diffraction information from conventional confocal datasets, broadening access to enhanced spatial resolution without specialised hardware.^158^

AI is increasingly integrated into adaptive microscopy systems capable of real‐time decision‐making during image acquisition.^159^ Such approaches enable the detection of rare trafficking events, automated tracking of vesicle dynamics, and optimisation of imaging parameters in response to cellular behaviour. These developments are particularly relevant for studying rapid ER‐Golgi interactions and transient vesicle fusion events. At a systems level, machine learning facilitates high‐content phenotypic profiling linking secretory pathway morphology to genetic perturbations, advancing quantitative and predictive models of trafficking.^160^ However, AI approaches introduce new challenges, including training bias, interpretability limitations, and dependence on high‐quality annotated datasets. Rigorous validation and transparency remain essential. Collectively, artificial intelligence is transforming microscopy from a predominantly observational discipline into a quantitative, data‐intensive science capable of generating mechanistic and predictive insight into secretory pathway organisation.

**FIGURE 6:**
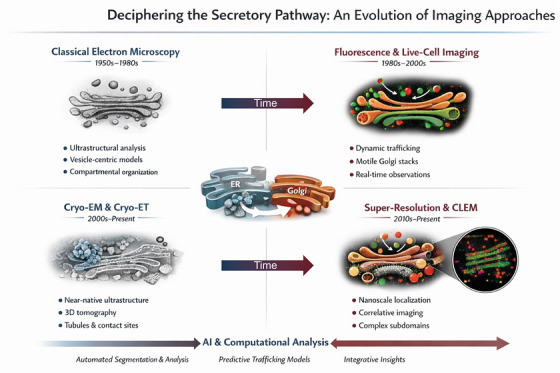
Deciphering the secretory pathway: evolution of imaging approaches across scales. Schematic overview illustrating how successive advances in microscopy have shaped conceptual models of the secretory pathway. Classical transmission electron microscopy (1950s–1980s) established the ultrastructural organisation of the ER‐Golgi system and supported vesicle‐centric models of compartmentalised transport. The fluorescence revolution (1980s–2000s), driven by genetically encoded reporters and live‐cell imaging, revealed dynamic organelle behaviour, cargo flux, and cytoskeleton‐dependent Golgi motility, particularly in plant cells. Cryo‐electron microscopy and cryo‐electron tomography (2000s–present) enabled near‐native three‐dimensional visualisation of membrane architecture, coat assemblies, and ER‐Golgi interfaces, refining structural models of transport intermediates. More recently, super‐resolution microscopy and correlative light and electron microscopy (2010s–present) have resolved nanoscale compartmentalisation and directly linked molecular identity to ultrastructure. Artificial intelligence and computational microscopy now integrate these modalities through automated segmentation, quantitative analysis, and predictive modelling, transforming the secretory pathway from a descriptive morphological system into a dynamic, multiscale, and increasingly quantitative framework.

In summary, the history of secretory pathway research demonstrates that advances in microscopy do more than improve resolution; they reshape conceptual models (Figure 6). Classical electron microscopy established the structural framework of the ER‐Golgi system and supported compartmental, vesicle‐centric interpretations of transport. Functional EM linked structure to biosynthetic activity but left kinetic questions unresolved. Live‐cell fluorescence imaging introduced temporal insight, revealing organelle motility and cargo flux that challenged static views and strengthened dynamic models such as cisternal maturation. In plant cells, highly motile Golgi stacks and close ER‐Golgi coupling further refine these interpretations.

Recent developments have added both precision and complexity. Cryo‐electron microscopy visualises near‐native architecture, constraining models of vesicle formation and membrane organisation. Super‐resolution imaging has uncovered nanoscale subdomains within organelles once considered uniform, while correlative approaches integrate molecular identity with ultrastructure. Rather than resolving longstanding debates outright, these methods have revealed additional layers of heterogeneity. Artificial intelligence and computational microscopy now enable large‐scale quantitative analysis, moving the field toward data‐driven and increasingly predictive frameworks. Together, integrative imaging strategies are transforming the secretory pathway into a multiscale system in which structure, dynamics, and molecular organisation can be interrogated in parallel. Yet fundamental mechanistic questions remain, ensuring that future advances in microscopy will continue not only to clarify but also to redefine, our understanding of intracellular trafficking.

## CONFLICT OF INTEREST STATEMENT

The authors declare no conflicts of interest.

## FUNDING INFORMATION

CP was supported by an Early Career Fellowship grant from the Leverhulme Trust (ECF‐2022‐002). EF was supported by funding from the Biotechnology and Biological Sciences Research Council (BB/T008784/1).
